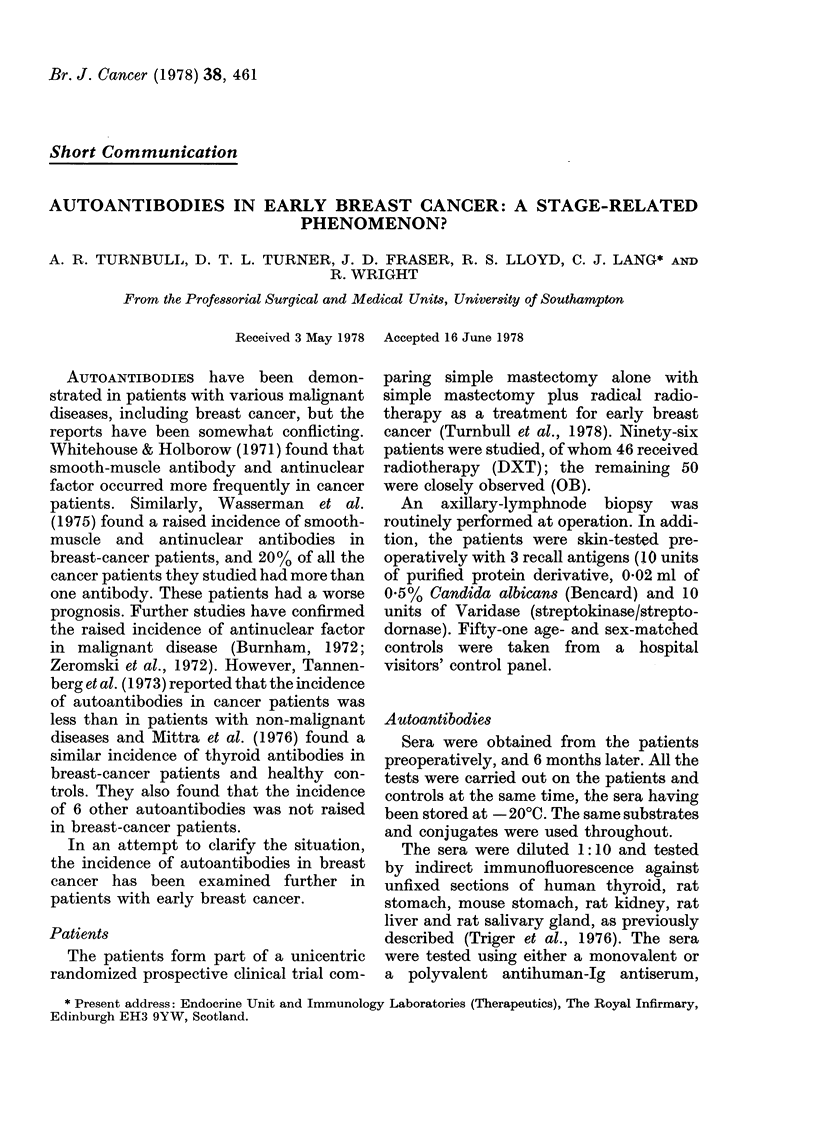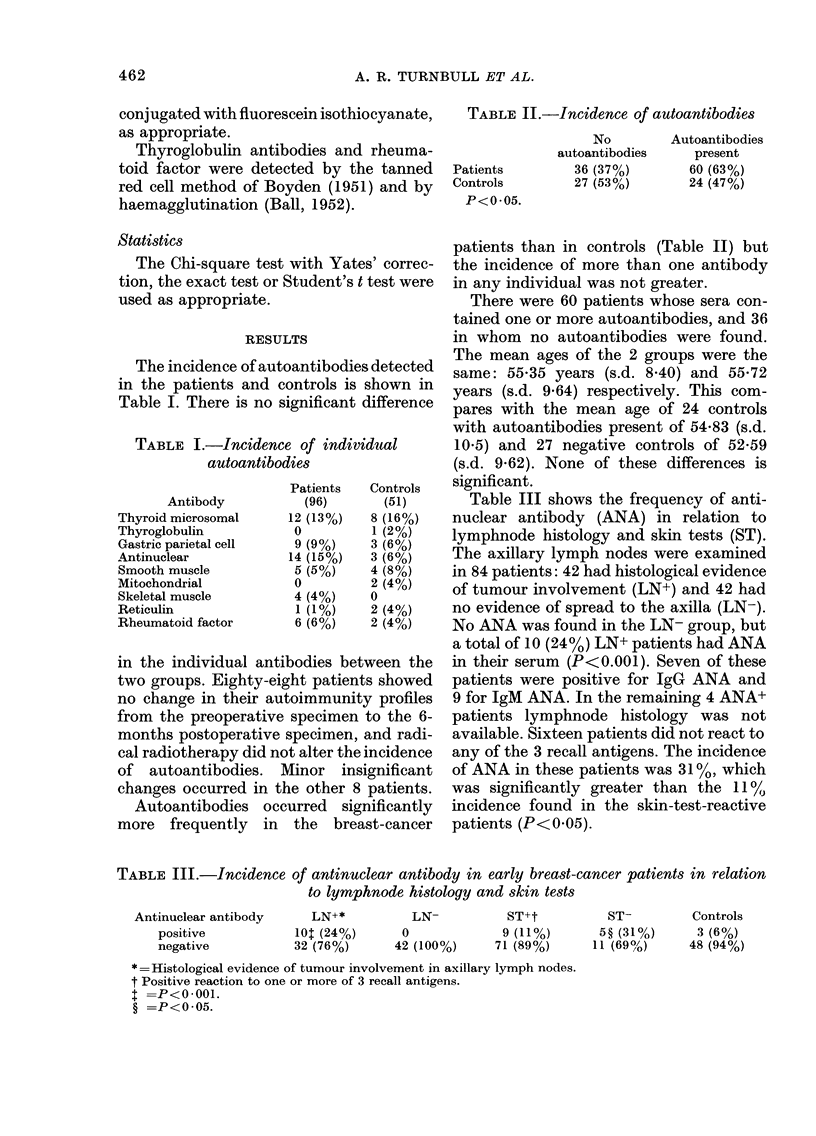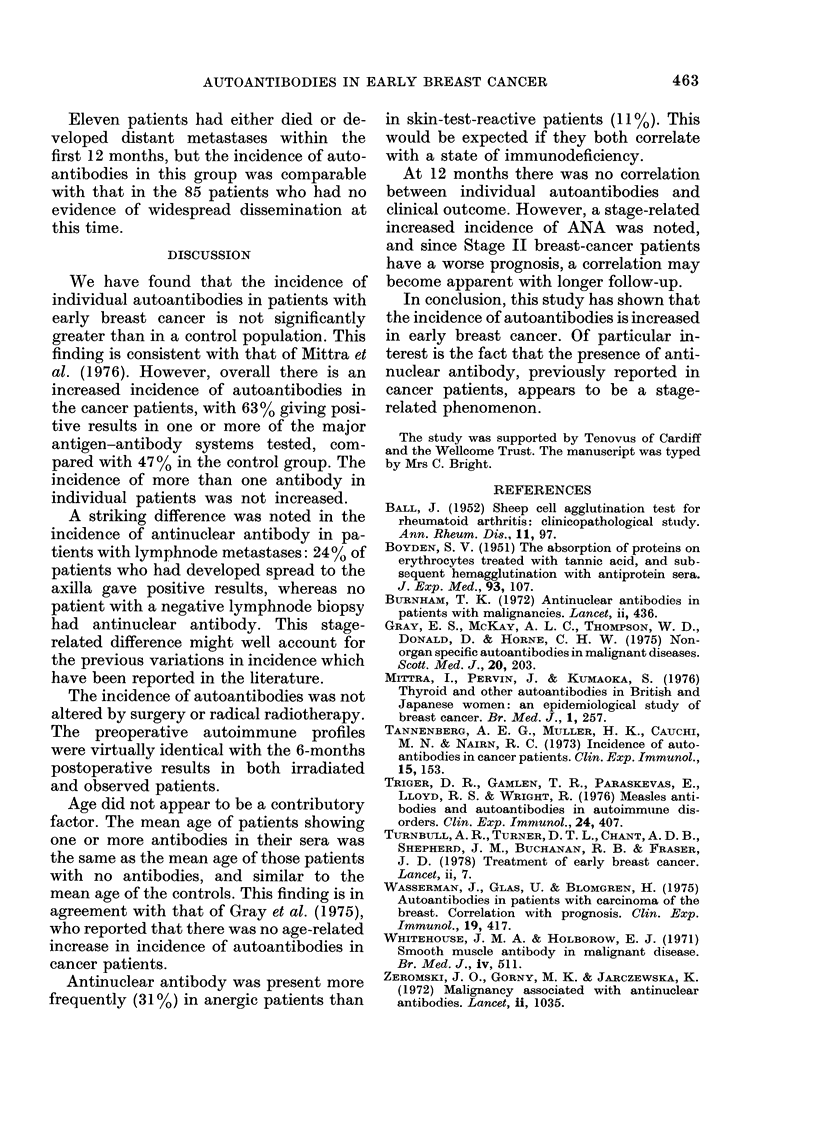# Autoantibodies in early breast cancer: a stage-related phenomenon?

**DOI:** 10.1038/bjc.1978.230

**Published:** 1978-09

**Authors:** A. R. Turnbull, D. T. Turner, J. D. Fraser, R. S. Lloyd, C. J. Lang, R. Wright


*Br. J. Cancer* (1978) **38**, 461

### *Short Communication*

### AUTOANTIBODIES IN EARLY BREAST CANCER: A STAGE-RELATED PHENOMENON?

A. R. TURNBULL, D. T. L. TURNER, J. D. FRASER, R. S. LLOYD, C. J. LANG* AND R. WRIGHT

*From the Professorial Surgical and Medical Units, University of Southampton*

Received 3 May 1978 Accepted 16 June 1978

AUTOANTIBODIES have been demonstrated in patients with various malignant diseases, including breast cancer, but the reports have been somewhat conflicting. Whitehouse & Holborow (1971) found that smooth-muscle antibody and antinuclear factor occurred more frequently in cancer patients. Similarly, Wasserman *et al.* (1975) found a raised incidence of smooth-muscle and antinuclear antibodies in breast-cancer patients, and 20% of all the cancer patients they studied had more than one antibody. These patients had a worse prognosis. Further studies have confirmed the raised incidence of antinuclear factor in malignant disease (Burnham, 1972; Zeromski *et al.*, 1972). However, Tannenberg *et al.* (1973) reported that the incidence of autoantibodies in cancer patients was less than in patients with non-malignant diseases and Mittra *et al.* (1976) found a similar incidence of thyroid antibodies in breast-cancer patients and healthy controls. They also found that the incidence of 6 other autoantibodies was not raised in breast-cancer patients.

In an attempt to clarify the situation, the incidence of autoantibodies in breast cancer has been examined further in patients with early breast cancer.

### *Patients*

The patients form part of a unicentric randomized prospective clinical trial comparing simple mastectomy alone with simple mastectomy plus radical radiotherapy as a treatment for early breast cancer (Turnbull *et al.*, 1978). Ninety-six patients were studied, of whom 46 received radiotherapy (DXT); the remaining 50 were closely observed (OB).

An axillary-lymphnode biopsy was routinely performed at operation. In addition, the patients were skin-tested preoperatively with 3 recall antigens (10 units of purified protein derivative, 0·02 ml of 0·5% *Candida albicans* (Bencard) and 10 units of Varidase (streptokinase/streptodornase). Fifty-one age- and sex-matched controls were taken from a hospital visitors' control panel.

### *Autoantibodies*

Sera were obtained from the patients preoperatively, and 6 months later. All the tests were carried out on the patients and controls at the same time, the sera having been stored at −20°C. The same substrates and conjugates were used throughout.

The sera were diluted 1:10 and tested by indirect immunofluorescence against unfixed sections of human thyroid, rat stomach, mouse stomach, rat kidney, rat liver and rat salivary gland, as previously described (Triger *et al.*, 1976). The sera were tested using either a monovalent or a polyvalent antihuman-Ig antiserum,

* Present address: Endocrine Unit and Immunology Laboratories (Therapeutics), The Royal Infirmary, Edinburgh EH3 9YW, Scotland.

conjugated with fluorescein isothiocyanate, as appropriate.

Thyroglobulin antibodies and rheumatoid factor were detected by the tanned red cell method of Boyden (1951) and by haemagglutination (Ball, 1952).

*Statistics*

The Chi-square test with Yates' correction, the exact test or Student's $t$ test were used as appropriate.

### RESULTS

The incidence of autoantibodies detected in the patients and controls is shown in Table I. There is no significant difference

TABLE I.—*Incidence of individual autoantibodies*

| Antibody | Patients (96) | Controls (51) |
|---|---|---|
| Thyroid microsomal | 12 (13%) | 8 (16%) |
| Thyroglobulin | 0 | 1 (2%) |
| Gastric parietal cell | 9 (9%) | 3 (6%) |
| Antinuclear | 14 (15%) | 3 (6%) |
| Smooth muscle | 5 (5%) | 4 (8%) |
| Mitochondrial | 0 | 2 (4%) |
| Skeletal muscle | 4 (4%) | 0 |
| Reticulin | 1 (1%) | 2 (4%) |
| Rheumatoid factor | 6 (6%) | 2 (4%) |

in the individual antibodies between the two groups. Eighty-eight patients showed no change in their autoimmunity profiles from the preoperative specimen to the 6-months postoperative specimen, and radical radiotherapy did not alter the incidence of autoantibodies. Minor insignificant changes occurred in the other 8 patients.

Autoantibodies occurred significantly more frequently in the breast-cancer

TABLE II.—*Incidence of autoantibodies*

| | No autoantibodies | Autoantibodies present |
|---|---|---|
| Patients | 36 (37%) | 60 (63%) |
| Controls | 27 (53%) | 24 (47%) |

$P < 0{\cdot}05$.

patients than in controls (Table II) but the incidence of more than one antibody in any individual was not greater.

There were 60 patients whose sera contained one or more autoantibodies, and 36 in whom no autoantibodies were found. The mean ages of the 2 groups were the same: 55·35 years (s.d. 8·40) and 55·72 years (s.d. 9·64) respectively. This compares with the mean age of 24 controls with autoantibodies present of 54·83 (s.d. 10·5) and 27 negative controls of 52·59 (s.d. 9·62). None of these differences is significant.

Table III shows the frequency of antinuclear antibody (ANA) in relation to lymphnode histology and skin tests (ST). The axillary lymph nodes were examined in 84 patients: 42 had histological evidence of tumour involvement ($LN^+$) and 42 had no evidence of spread to the axilla ($LN^-$). No ANA was found in the $LN^-$ group, but a total of 10 (24%) $LN^+$ patients had ANA in their serum ($P < 0.001$). Seven of these patients were positive for IgG ANA and 9 for IgM ANA. In the remaining 4 $ANA^+$ patients lymphnode histology was not available. Sixteen patients did not react to any of the 3 recall antigens. The incidence of ANA in these patients was 31%, which was significantly greater than the 11% incidence found in the skin-test-reactive patients ($P < 0{\cdot}05$).

TABLE III.—*Incidence of antinuclear antibody in early breast-cancer patients in relation to lymphnode histology and skin tests*

| Antinuclear antibody | $LN^+$* | $LN^-$ | $ST^+$† | $ST^-$ | Controls |
|---|---|---|---|---|---|
| positive | 10‡ (24%) | 0 | 9 (11%) | 5§ (31%) | 3 (6%) |
| negative | 32 (76%) | 42 (100%) | 71 (89%) | 11 (69%) | 48 (94%) |

* = Histological evidence of tumour involvement in axillary lymph nodes.
† Positive reaction to one or more of 3 recall antigens.
‡ $= P < 0{\cdot}001$.
§ $= P < 0{\cdot}05$.

Eleven patients had either died or developed distant metastases within the first 12 months, but the incidence of autoantibodies in this group was comparable with that in the 85 patients who had no evidence of widespread dissemination at this time.

### DISCUSSION

We have found that the incidence of individual autoantibodies in patients with early breast cancer is not significantly greater than in a control population. This finding is consistent with that of Mittra *et al.* (1976). However, overall there is an increased incidence of autoantibodies in the cancer patients, with 63% giving positive results in one or more of the major antigen–antibody systems tested, compared with 47% in the control group. The incidence of more than one antibody in individual patients was not increased.

A striking difference was noted in the incidence of antinuclear antibody in patients with lymphnode metastases: 24% of patients who had developed spread to the axilla gave positive results, whereas no patient with a negative lymphnode biopsy had antinuclear antibody. This stage-related difference might well account for the previous variations in incidence which have been reported in the literature.

The incidence of autoantibodies was not altered by surgery or radical radiotherapy. The preoperative autoimmune profiles were virtually identical with the 6-months postoperative results in both irradiated and observed patients.

Age did not appear to be a contributory factor. The mean age of patients showing one or more antibodies in their sera was the same as the mean age of those patients with no antibodies, and similar to the mean age of the controls. This finding is in agreement with that of Gray *et al.* (1975), who reported that there was no age-related increase in incidence of autoantibodies in cancer patients.

Antinuclear antibody was present more frequently (31%) in anergic patients than in skin-test-reactive patients (11%). This would be expected if they both correlate with a state of immunodeficiency.

At 12 months there was no correlation between individual autoantibodies and clinical outcome. However, a stage-related increased incidence of ANA was noted, and since Stage II breast-cancer patients have a worse prognosis, a correlation may become apparent with longer follow-up.

In conclusion, this study has shown that the incidence of autoantibodies is increased in early breast cancer. Of particular interest is the fact that the presence of antinuclear antibody, previously reported in cancer patients, appears to be a stage-related phenomenon.

The study was supported by Tenovus of Cardiff and the Wellcome Trust. The manuscript was typed by Mrs C. Bright.

### REFERENCES

Ball, J. (1952) Sheep cell agglutination test for rheumatoid arthritis: clinicopathological study. *Ann. Rheum. Dis.*, **11**, 97.

Boyden, S. V. (1951) The absorption of proteins on erythrocytes treated with tannic acid, and subsequent hemagglutination with antiprotein sera. *J. Exp. Med.*, **93**, 107.

Burnham, T. K. (1972) Antinuclear antibodies in patients with malignancies. *Lancet*, ii, 436.

Gray, E. S., McKay, A. L. C., Thompson, W. D., Donald, D. & Horne, C. H. W. (1975) Non-organ specific autoantibodies in malignant diseases. *Scott. Med. J.*, **20**, 203.

Mittra, I., Pervin, J. & Kumaoka, S. (1976) Thyroid and other autoantibodies in British and Japanese women: an epidemiological study of breast cancer. *Br. Med. J.*, **1**, 257.

Tannenberg, A. E. G., Muller, H. K., Cauchi, M. N. & Nairn, R. C. (1973) Incidence of autoantibodies in cancer patients. *Clin. Exp. Immunol.*, **15**, 153.

Triger, D. R., Gamlen, T. R., Paraskevas, E., Lloyd, R. S. & Wright, R. (1976) Measles antibodies and autoantibodies in autoimmune disorders. *Clin. Exp. Immunol.*, **24**, 407.

Turnbull, A. R., Turner, D. T. L., Chant, A. D. B., Shepherd, J. M., Buchanan, R. B. & Fraser, J. D. (1978) Treatment of early breast cancer. *Lancet*, ii, 7.

Wasserman, J., Glas, U. & Blomgren, H. (1975) Autoantibodies in patients with carcinoma of the breast. Correlation with prognosis. *Clin. Exp. Immunol.*, **19**, 417.

Whitehouse, J. M. A. & Holborow, E. J. (1971) Smooth muscle antibody in malignant disease. *Br. Med. J.*, **iv**, 511.

Zeromski, J. O., Gorny, M. K. & Jarczewska, K. (1972) Malignancy associated with antinuclear antibodies. *Lancet*, **ii**, 1035.